# Determining a Clinically Relevant Strategy for Bone Tissue Engineering: An “All-in-One” Study in Nude Mice

**DOI:** 10.1371/journal.pone.0081599

**Published:** 2013-12-11

**Authors:** Pierre Corre, Christophe Merceron, Caroline Vignes, Sophie Sourice, Martial Masson, Nicolas Durand, Florent Espitalier, Paul Pilet, Thomas Cordonnier, Jacques Mercier, Séverine Remy, Ignacio Anegon, Pierre Weiss, Jérôme Guicheux

**Affiliations:** 1 INSERM (Institut National de la Santé et de la Recherche Médicale), UMR (Unité Mixte de Recherche) 791, center for osteoarticular and dental tissue engineering, Université de Nantes, Nantes, France; 2 Centre Hospitalier Universitaire de Nantes, Clinique de Stomatologie et de Chirurgie maxillo-faciale, Nantes, France; 3 Centre Hospitalier Universitaire de Nantes, Clinique d'Oto-Rhino-Laryngologie et de Chirurgie cervico-faciale, Nantes, France; 4 INSERM, UMR 1064, Centre pour la recherche en transplantation et immunologie et Plate-forme Transgenic Rats Nantes, Institut de Transplantation Urologie-Néphrologie (ITUN), Nantes, France; 5 Centre Hospitalier Universitaire de Nantes, Pôle Hospitalo-Universitaire 4, Nantes, France; Université de Technologie de Compiègne, France

## Abstract

**Purpose:**

Autologous bone grafting (BG) remains the standard reconstruction strategy for large craniofacial defects. Calcium phosphate (CaP) biomaterials, such as biphasic calcium phosphate (BCP), do not yield consistent results when used alone and must then be combined with cells through bone tissue engineering (BTE). In this context, total bone marrow (TBM) and bone marrow-derived mesenchymal stem cells (MSC) are the primary sources of cellular material used with biomaterials. However, several other BTE strategies exist, including the use of growth factors, various scaffolds, and MSC isolated from different tissues. Thus, clinicians might be unsure as to which method offers patients the most benefit. For this reason, the aim of this study was to compare eight clinically relevant BTE methods in an “all-in-one” study.

**Methods:**

We used a transgenic rat strain expressing green fluorescent protein (GFP), from which BG, TBM, and MSC were harvested. Progenitor cells were then mixed with CaP materials and implanted subcutaneously into nude mice. After eight weeks, bone formation was evaluated by histology and scanning electron microscopy, and GFP-expressing cells were tracked with photon fluorescence microscopy.

**Results/Conclusions:**

Bone formation was observed in only four groups. These included CaP materials mixed with BG or TBM, in which abundant *de novo* bone was formed, and BCP mixed with committed cells grown in two- and three-dimensions, which yielded limited bone formation. Fluorescence microscopy revealed that only the TBM and BG groups were positive for GFP expressing-cells, suggesting that these donor cells were still present in the host and contributed to the formation of bone. Since the TBM-based procedure does not require bone harvest or cell culture techniques, but provides abundant *de novo* bone formation, we recommend consideration of this strategy for clinical applications.

## Introduction

Bone reconstruction might be required in patients with craniofacial defects (e.g., craniofacial trauma or malformations) or following resection of oral carcinomas. Autologous bone grafting (BG) is considered the gold standard in bone reconstruction [1]. However, the harvesting procedure requires a second surgical site where potential complications have been reported [2], [3]. Moreover, allografts and xenografts are sometimes problematic as they are limited in supply and at risk for cross-contamination [4], [5].

Calcium phosphate (CaP) biomaterials, and particularly biphasic calcium phosphate (BCP), have shown clinical efficacy in orthopedic surgery [6], [7], avoiding second surgical site morbidity and reducing surgery time compared to autologous grafting [7], [8]. However, although CaP biomaterials contribute to bone healing through osteoconduction, they generally lack osteoinductivity for regenerating large bone tissue defects, or in tissues exposed to sources of infection (i.e., oronasal mucosa), because they have little contact with bone. Therefore, clinical applications must be restricted to small bone defects or to regions with significant bone contact [9]. In view of these limitations, surgeons and researchers have focused on developing alternative therapies to BG over the past fifteen years. These mainly included combining osteoprogenitor cells with bone substitutes to improve their osteogenic properties. Thus, extemporaneous mixtures of CaP biomaterials and unprocessed total fresh bone marrow (TBM) have shown osteogenic potential *in vivo*. Indeed, in rat models, this technique successfully induced bone formation in extraosseous sites [10], [11] and potentiated bone ingrowth in osseous sites [12], [13]. Moreover, the efficacy of CaP biomaterials (i.e., granules, sticks, or blocks) combined with autologous bone marrow or autologous cancellous bone has been shown in clinical studies for defect filling in various orthopedic procedures [14].

More recently, the use of mesenchymal stem cells (MSC) isolated from bone marrow and combined with various scaffolds has emerged as a potential new treatment strategy. Indeed, this bone tissue engineering (BTE) technique showed promising results in small animal models [15], [16], as well as in orthotopic and ectopic clinically sized implants [17], [18]. However, the efficacy of BTE in humans has not yet been clinically validated [19]–[21]. Also, to improve the ability of engineered bone to replace autologous BG, new BTE strategies have been developed in recent years, including three-dimensional cell culture in bioreactors [22] and gene therapy. However, preclinical studies aimed at comparing the relative efficiency of these strategies have not been performed to date, and it remains difficult for clinicians to determine which method will offer patients the most benefit. Also, a major concern related to procedures involving MSC is the fate of cells after they enter the host via massive implantation. It remains unknown whether these MSC remain at the grafted site and are specifically responsible for new bone formation, as suggested by some authors [23], [24], or if they die early after implantation and release mediators for cell recruitment from the local environment, as suggested in more recent studies [25]–[27].

Therefore, the aim of the present study was to address some of the uncertainties regarding the clinical use of BTE. Specifically, in an “all-in-one” study, we compared the ectopic bone formation potential of eight well-known bone repair strategies using BCP granules and progenitor cells. Moreover, we assessed if complex BTE strategies were more efficient than *in vivo* BTE procedures, such as TBM combined with CaP biomaterials. In addition, green fluorescent protein (GFP) tracking was used to determine the fate of grafted cells after implantation [28].

## Materials and Methods

All procedures involving animals were conducted in accordance with the institutional guidelines of the French Ethical Committee (CEEA.PdL.06): their housing in the Experimental Therapeutic Unit at the Faculty of Medicine of Nantes (France), animal care, the method by which they were anesthetized and sacrificed, and all experimental protocols. All efforts were made to minimize suffering.

The European Community guidelines for the care and use of laboratory animals (DE 86/609/CEE, modified DE 2003/65/CE) have been revised by the new European directive 2010 (DE 2010/63/UE modified 22/09/2010). The translation of the EU regulation into the French one is effective from January 1st 2013. Submission of the project to the new Ethical Committee of the “Pays de la Loire” was not mandatory until January 1st 2013. Nevertheless, all the experiments were conducted prior to January 2013 accordingly to this new regulation.

The Veterinary Service (Center for Preclinical Research and Investigation of the ONIRIS Nantes-Atlantic College of Veterinary Medicine, Food Science and Engineering) approved the senior researcher in charge of the experiments.

### Materials

Plastic ware for cell culture was purchased from Corning (Schipol-Rijk, the Netherlands). Sodium L-ascorbate, vitamin D3, dexamethasone, Alizarin Red S, ITS media supplement, 3-isobutyl-1-methylxanthine (IBMX), indomethacin, Oil Red O, and trypan blue were purchased from Sigma-Aldrich (St. Louis, MO). Alpha minimum essential medium (αMEM), phosphate-buffered saline (PBS), penicillin-streptomycin, trypsin-EDTA (0.05%–0.53 mM), L-glutamine, Trizol^®^ reagent, and the superscript III kit were obtained from Invitrogen (Paisley, UK). Fetal calf serum (FCS) was provided by Dominique Dutscher (Brumath, France). β-glycerophosphate was purchased from Calbiochem (Darmstadt, Germany). Brilliant SYBR^®^ Green Master Mix was obtained from Stratagene Europe (Amsterdam Zuidoost, the Netherlands). Polymerase chain reaction (PCR) primers were synthesized by MWG Biotech (Ebersberg, Germany), and Turbo DNase was purchased from Ambion Inc. (distributed by Applied Biosystems; Courtaboeuf, France). All other chemicals were obtained from standard laboratory suppliers and were of the highest purity available.

### CaP biomaterials

Biphasic CaP particles made of hydroxyapatite (60%) and beta-tricalcium phosphate (40%) with a size between 500 and 1000 µm (MBCP^™^) were provided by Biomatlante (Vigneux de Bretagne, France). Tubes containing each 0.07 g of granules were double-packed and sterilized at 121°C for 20 min in an autoclave.

### Animals

Twenty-four female nude mice (S/SOPF SWISS; six weeks old) were obtained from a certified breeding center (C. River, l′Arbresle, France) and acclimatized for two weeks to the conditions of the local vivarium (24°C and a 12/12 h light/dark cycle).

In addition, three adult, male, GFP-transgenic rats, previously described [28], were obtained from the transgenic rat facility (INSERM, UMRS 1064, Nantes, France) and specially designated as bone marrow (BM), BG, and MSC donors. Briefly, these GFP-transgenic rats are generated by a lentiviral vector expressing GFP driven by the ubiquitous PGK promoter. These rats strongly express GFP in multiple tissues and in important cell types, especially in mature neurons, in leukocytes subtypes including myeloid and plasmacytoid dendritic cell and in bone marrow where nearly 80% of MSC are GFP-positive.

### Bone marrow harvesting

BM was harvested after sacrifice. Animals were anesthetized using in inhaled isoflurane (Forene^®^, Abott, Rungis, France) and sacrificed by an intracardiac overdose of sodium thiopental (Nesdonal^®^, Rhône-Merieux, France). Each femur or tibia was flushed with 1 mL of saline. The same rinse (1 mL) was used for all bones (i.e. 6 femurs and 6 tibias) in order to obtain the most concentrated TBM and to minimize the risk of inter-individual variation of BM components. The BM obtained from the 3 donors was immediately and aseptically transferred to heparinized tubes (Venoject II, Terumo Europe, Louvain, Belgium). Then about 300 µL have been used for *in vivo* implantation, corresponding to 30–50 µl per one construct (70 mg of BCP). The remained 700 µL were used for cytological analysis (∼200 µL), performed as previously described [29] and for BM culture (∼500 µL).

### Cancellous bone harvesting

Cancellous bone was harvested with a dental curette from the epiphysis of bone previously cut for BM harvesting. About 30–40 mg of cancellous bone were harvested from each bone (femur or tibia) allowing a combination of ∼70 mg of bone roughly crushed with 70 mg BCP granules per construct in a 1∶1 weight ratio. The bone was immediately mixed with BCP and implanted subcutaneously in the following 30 minutes.

### Isolation and expansion of bone marrow-derived mesenchymal stem cells

A portion of the total harvested BM volume was filtered through a 70 µm nylon mesh filter. MSC were then harvested from BM donors and expanded *in vitro* for about six weeks prior to implantation. The BM was seeded in a 75 cm^2^ treated polystyrene culture and MSC were isolated based on their adherence capacity after two days. Cells were then grown in proliferative medium, consisting of αMEM supplemented with 1% L-glutamine, 1% penicillin/streptomycin, and 10% FCS, and incubated at 37°C in a humidified atmosphere (95% air, 5% CO_2_). The medium was renewed twice weekly until cells were 80–90% confluent. Cells were then detached enzymatically from plastic by an incubation of 3–4 min with 0.25% trypsin/EDTA and counted with a Malassez hemocytometer using trypan blue exclusion dye. To obtain a large number of cells, MSC were further expanded in treated polystyrene culture flasks. All cells used in experiments were between passages 2 and 6.

### Osteogenic differentiation

#### Culture

For *in vitro* osteogenic differentiation of MSC, cells were seeded at a density of 1.10^4^ cells/cm^2^ in six-well plates and grown in the presence of proliferative or osteogenic medium (OM) for 14 and 28 days as described previously [30]. OM was composed of the proliferative medium supplemented with 10 mM β-glycerophosphate, 50 µM sodium L-ascorbate, and 10 nM vitamin D3. MSC were maintained at 37°C in a humidified atmosphere (5% CO_2_ and 95% air), and the media was changed every 2–3 days.

#### Calcium deposition

Calcium deposition was detected at 14 and 28 days by Alizarin Red S staining as described previously [31]. Briefly, MSC were grown as described above and washed with cold PBS followed by staining with 2% Alizarin Red S solution for 2 min. Stained cells were then extensively washed with deionized water to remove any nonspecific precipitates. Stained layers were visualized with phase microscopy using an inverted microscope (Nikon Eclipse TE 2000 E, Badhoevedorp, the Netherlands). Positive red staining indicated the deposition of a calcified matrix on the differentiated cells. Osteogenic differentiation was further characterized through the relative expression of osteogenic marker genes (*Alpl, Bglap*, and *Runx2*; see real-time PCR section below).

### Adipogenic differentiation

#### Culture

For *in vitro* adipogenic differentiation of MSC, cells were seeded at a density of 3.10^4^ cells/cm^2^ in six-well plates and grown in the presence of control or adipogenic medium for 14 days as previously described [30]. Adipogenic medium was composed of the control medium supplemented with 1 µM dexamethasone, 200 µM indomethacin, 0.5 mM IBMX, and ITS (10 µg/mL insulin; 10 µg/mL transferrin; 10 ng/mL sodium selenite). MSC were maintained at 37°C in a humidified atmosphere (5% CO_2_ and 95% air), and media was changed every 2–3 days.

#### Detection of neutral lipid droplets

Adipogenesis of BM-MSC was assessed by Oil Red O staining for lipid droplet detection. BM-MSC were grown as described above, washed with ice-cold PBS, and then fixed with 10% formalin for 5 min (followed by 1 h with fresh formalin at room temperature). The formalin was then discarded, and the wells were rinsed with 60% isopropanol. The wells were left to completely dry, and then 0.35% Oil Red O solution in 60% isopropanol was added for 10 min. Stained cells were extensively washed with deionized water to remove any nonspecific staining. Samples were visualized using a light microscope (Zeiss Axioplan 2, Oberkochen, Germany), and red staining indicated the presence of neutral lipid droplets. Adipogenic differentiation was also characterized through the relative expression of adipogenic marker genes (including *Pparγ* and *Lep*; see real-time PCR section below).

#### Real-time polymerization chain reaction

For real-time PCR analysis, total RNA was extracted using TRIzol^®^ according to the manufacturer's instructions. After DNase I digestion, RNA was quantified with a UV-spectrophotometer (Nanodrop ND-1000, Labtech, Palaiseau, France) and quality was determined with the Agilent Bioanalyzer 2100 system (Waldbronn, Germany). A total of 500 ng of RNA per sample were reverse transcribed using the superscript III kit in a total volume of 30 µL. Complementary DNA (cDNA) was amplified in a 25 µL (total volume) PCR reaction mix containing 12.5 µL of Brilliant SYBR Green Master Mix^®^ and 30 nM of SYBR green reference dye. The sequences of each primer set are provided in Table 1. The real-time PCR was carried out in a MX3000P real-time PCR system (Stratagene) under the following conditions: 10 min at 95°C followed by 40 successive cycles of 30 s at 95°C, 1 min at 60°C, and 30 s at 72°C. The efficiency and specificity of each primer set were confirmed with standard curves of cycle threshold (Ct) values vs. serial dilution of total RNA and melting profile evaluation. Ct values were normalized to β-actin as a housekeeping gene to control for differences in cDNA quantification. Results were reported as relative expression levels.

**Table 1:** Gene names and abbreviations, gene bank accession numbers, sequences of primer pairs (Fwd: Forward, Rev: Reverse) and length of PCR products used for real-time RT-PCR analysis.

Gene	Gene Bank	Sequence	Base Pairs (bp)
	Accession Number		
β-Actin	031144.2	Fwd 5′- cccgcgagtacaaccttct –3′	72
(*Actb*)		Rev 5′- cgtcatccatggcgaac –3′	
Alkaline phosphatase,	013059.1	Fwd 5′- gcacaacatcaaggacatcg –3′	72
liver/bone/kidney		Rev 5′- tcagttctgttcttggggtacat –3′	
(*Alpl*)			
Bone gamma-	013414.1	Fwd 5′- atagactccggcgctacctc –3′	63
carboxyglutamate (gla)		Rev 5′- ccaggggatctgggtagg –3′	
protein ( = Osteocalcin)			
(*Bglap*)			
runt-related transcription	053470.2	Fwd 5′- cacagagctattaaagtgacagtgg -3′	86
factor 2		Rev 5′- aacaaactaggtttagagtcatcaagc – 3′	
(*Runx2*)			
Peroxisome proliferator-	013124.2	Fwd 5′- ggtgaaactctgggagatcct - 3′	110
activated receptor gamma		Rev 5′- aatggcatctctgtgtcaacc –3′	
(*Pparγ*)			
Leptin	013076.2	Fwd 5′- ccaggatcaatgacatttcaca –3′	71
(*Lep*)		Rev 5′- aatgaagtccaaaccggtga –3′	

### Cell culture on biphasic calcium phosphate particles

Cells were seeded on BCP granules and grown for two weeks as previously described [32]. Briefly, to minimize ion release from the BCP particles during cell culture, particles were incubated 48 h in twice-refreshed control medium. Prior to culture, MSC were loaded on the BCP particles at a density of 9.10^4^ cells/cm^2^ of BCP, corresponding to 7.5.10^3^ cells/mg of BCP as previously described [33]. To favor cell adhesion on ceramic particles, the culture was performed in low attachment 24-well plates. Cells were then grown during 14 days in osteogenic or proliferative medium. The culture media were refreshed every 2–3 days. Osteogenic differentiation was characterized through the relative gene expression of osteogenic marker genes (*Alpl, Bglap*, and *Runx2*).

### Cell attachment, viability and osteogenic differentiation onto the CaP biomaterials

For the MSC grown on BCP granules in OM and PM, cell attachment was analyzed at day 14 using a methylene blue staining (Certistain, Merck, Darmstadt, Germany) and scanning electron microscopy (SEM). For methylene blue staining, samples were evaluated by light microscopy. For SEM, samples were dehydrated with graded ethanol, fixed with trichlorotrifluoroethane, and then sputtered with a thin layer of gold-palladium (EM Scope, England). Three samples from each group (osteogenic or control medium) were analyzed by SEM (LEO VP1450, Zeiss, Oberkochen, Germany). Cell viability at Day 14 was assessed by tracking GFP-expressing cells on the biomaterial surface using a Nikon Eclipse TE 2000 E microscope (Badhoevedorp, the Netherlands).

### Implant preparation

**Figure 1:**
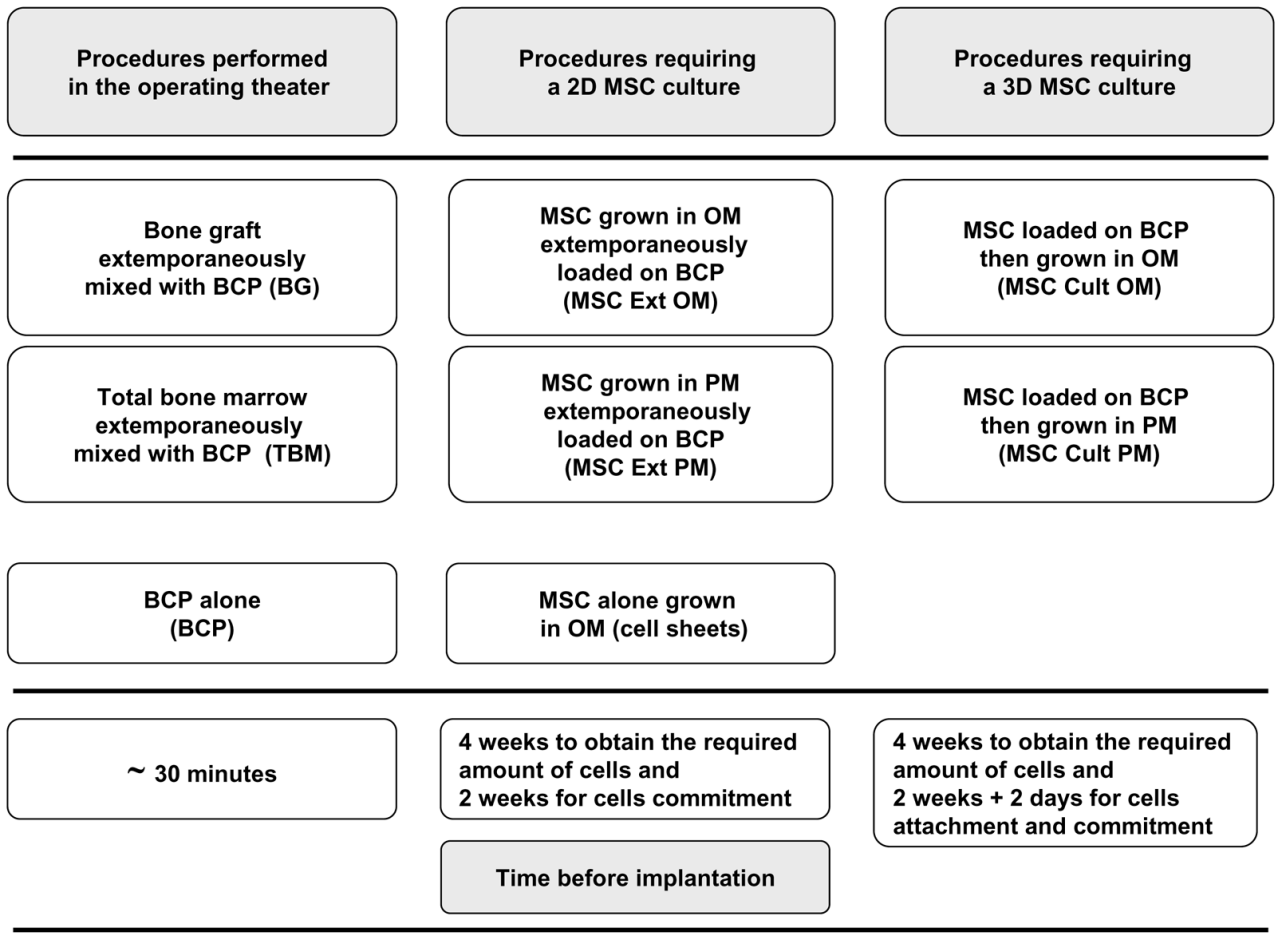
Outline of the protocol illustrating the different procedures and the required time before implantation for each condition. OM: osteogenic medium, PM: proliferative medium, 2D: two-dimensional, 3D: three-dimensional, MSC: mesenchymal stem cells, BCP: biphasic calcium phosphate, Ext: extemporaneously, Cult: cultured.

Eight groups of implants were prepared (Fig. 1), thus reproducing eight bone repair procedures that previously demonstrated *in vivo* osteogenic properties in animal models or humans [14], [34]–[39]:

1. BG extemporaneously combined with BCP granules (BG).
2. CaP granules alone (BCP).
3. Total bone marrow graft extemporaneously combined with BCP granules (TBM).
4. MSC differentiated in OM extemporaneously loaded on BCP granules (MSC Ext OM).
5. MSC grown in proliferative medium extemporaneously loaded on BCP (MSC Ext PM).
6. MSC grown on BCP granules in OM (MSC Cult OM).
7. MSC grown on BCP granules in proliferative medium (MSC Cult PM).
8. Osteoprogenitor cell sheet transplantation using hyperconfluent cells (seeded at 5.10^4^ cells/cm^2^ and grown during 14 days in OM) (Cell sheets).

For extemporaneous mixing procedures (BG, TBM, MSC Ext OM, and MSC Ext PM), cell preparations were allowed to attach to the ceramics for at least 30 min at room temperature prior to implantation. For MSC Ext OM and MSC Ext PM conditions, MSC were loaded on BCP particles at a density of 9.10^4^ cells/cm^2^ of BCP (∼7.5.10^3^ cells/mg) as previously described [33]. An overview of the combination of cells and scaffold is provided in table 2.

**Table 2:** Cells and material combination related to the experimental groups.

Experimental condition	Mass of material per implant (volume)	Density of seeded MSC per mg of material (or per cm^2^ of material)	Mass of BG per implant	Volume of TBM per implant
BG	70 mg (∼100 µl)	-	∼70 mg (∼100 µl)	-
BCP	70 mg (∼100 µl)	-	-	-
TBM	70 mg (∼100 µl)	-	-	∼30–50 µl
MSC Ext OM	70 mg (∼100 µl)	∼7.5.10^3^ cells/mg (∼9.10^4^ cells/cm^2^)	-	-
MSC Ext PM	70 mg (∼100 µl)	∼7.5.10^3^ cells/mg (∼9.10^4^ cells/cm^2^)	-	-
MSC Cult OM	70 mg (∼100 µl)	∼7.5.10^3^ cells/mg (∼9.10^4^ cells/cm^2^)	-	-
MSC Cult PM	70 mg (∼100 µl)	∼7.5.10^3^ cells/mg (∼9.10^4^ cells/cm^2^)	-	-
Cell sheets	-	∼5.10^4^ cells/cm^2^ of culture dish	-	-

MSC =  mesenchymal stem cells, BG =  bone graft, TBM =  Total bone marrow, MSC Ext OM/MSC Ext PM  =  Mesenchymal stem cells grown in osteogenic medium/proliferative medium then extemporaneously loaded onto BCP granules. MSC Cult OM/MSC Cult PM  =  MSC loaded onto BCP granules then grown in osteogenic/proliferative medium. BCP =  biphasic calcium phosphate alone.

### Surgical procedure

All surgical procedures were performed under general anesthesia (4% isoflurane inhalation for induction and 2% for preservation), and the samples were implanted subcutaneously in the backs of 24 nude mice. Briefly, a 1-cm transverse incision was made bilaterally along the dorsum of the back. Blunt dissection was performed to separate the skin from the subcutaneous connective tissues and to form several pockets under the skin into which implants were inserted. Each mouse received two randomly assigned implants (n = 6). Immediate postoperative analgesia was provided through subcutaneous injection of buprenorphine hydrochloride (Buprecare^®^ 0.3 mg/mL, 10 µg/kg, ANIMALCARE, Dunnington, UK), and maintained for 2 days. Implants were removed right after sacrifice via CO_2_ overdose, eight weeks after implantation.

### Scanning electron microscopy

The explanted specimens were fixed for 24 h in a 4% paraformaldehyde in phosphate-buffered saline, and then dehydrated through a graded series of ethanol treatments. Non-decalcified bone specimens were infiltrated and embedded in glycol-methyl-methacrylate (GMMA) obtained by mixing methyl methacrylate (Prolabo, Paris, France), polyethylene glycol 400 (Prolabo, Paris, France), benzoyl peroxide (Merck, Darmstadt, Germany). Polymerization was started with N-N-dimethyl-aniline (Sigma Aldrich, Saint-Quentin Fallavier, France) at 4°C for 72 h. Samples were cut perpendicularly from the implant using a circular diamond saw (Leica, SP1600, Wetzlar, Germany) and then sanded on a Metaserv 2000 (Buehler, Lake Bluff, USA) then gold-palladium-coated on a Desk III (Denton Vacuum, Moorestown, USA). SEM micrographs were taken using backscattered electrons at 15 kV. The surface of the implant was divided into contiguous high-resolution images, and the evaluation was performed with a semiautomatic image analyzer (Leica Quantimeter 500, Cambridge, UK). First, the contours of the defects were traced, then areas of newly formed mineralized bone, BCP granules, and non-mineralized tissues were identified by their grey levels.

### Histological assessment

For each sample, serial 5-µm thick sections were cut perpendicularly from the implant using a circular diamond saw (Leica, SP1600, Wetzlar, Germany) and a hard tissue microtome (Leica Polycut SM 2500, Wetzlar, Germany). The sections were stained with Goldner's trichrome and examined with a light microscope (Zeiss, axioplan2, Darmstadt, Germany). Semi-quantitative histological assessment of the amount of bone formed within the pores of the scaffolds was performed on the 6 samples per group as previously described [40]. Three sections, cut approximately 0.75 mm apart depth-wise, were analyzed per sample. A score from 0 to 4 was attributed to each section (Table 3). The mean score per sample was determined. For each section, randomized scoring was performed by two well-versed independent examiners (n = 3 per replicate).

**Table 3:** Semi-quantitative histological scoring system.

Score Description	
0	No bone
1	1–25% of available pore space filled with bone
2	26–50% of available pore space filled with bone
3	51–75% of available pore space filled with bone
4	76–100% of available pore space filled with bone

### “In vivo” tracking of donor cells by confocal microscopy

GFP expression was first carefully checked in all cell preparations at each of the steps of the study, including after TBM harvest, during cell amplification, and during osteogenic/adipogenic differentiation. Fluorescent cells were examined using a Nikon Eclipse TE 2000 E microscope and images were captured with a digital camera (Cool Snap EZ, Photometrics, Roper Scientific Molecular Devices, Evry, France) using NIS Element Nikon v.3.10. Finally, GFP-expressing cells were tracked *in vivo* in the GMMA-embedded implants. The histological sections were mounted under cover slips with the Prolong Gold Antifade reagent (Molecular Probes, Eugene, OR) and visualized with the same microscope. Image processing was performed using NIKON EZ C1 v.3.60. Wildtype BM from non GFP-expressing adult rats (INSERM, UMRS 1064, Nantes, France) was used as control for “*in vitro*” and “*in vivo*” tracking before and after implantation.

### Statistical analysis

Results were expressed as mean ± SD of six samples. Means were then compared using one-way ANOVA followed by a post-hoc test (Fisher's protected least significant difference), and p-values <0.05 were considered statistically significant.

## Results

### BM myelography

Cytology and myelography showed that the BM contained a normal amount of cells (51.10^6^ cells/mm^3^). The marrow cell lineages were analyzed (i.e., myeloblasts, myelocytes, proerythroblasts, erythroblasts, megakaryocytes, lymphocytes, plasmocytes, and monocytes). The results (Table 4) confirmed that the harvested BM contained physiologically relevant cell lineages but no blood. GFP-expressing cells were tracked after TBM harvesting. The BM exhibited about 80% of GFP positive cells, while non-GFP wildtype BM did not exhibit any fluorescence (data not shown).

**Table 4:** Cytological analysis of the bone marrow. Results are expressed as a percentage of cells per lineage.

Granulocytic lineages		Erythroblastic	Megacaryocytic	Lymphocyte	Plasmocyte	Monocyte	Blasts
Myeloblastic	Myelocytic	lineages	lineage				
1.5	26	32.5	Physiological	35	0	1	4

### Multidifferentiation potential

**Figure 2:**
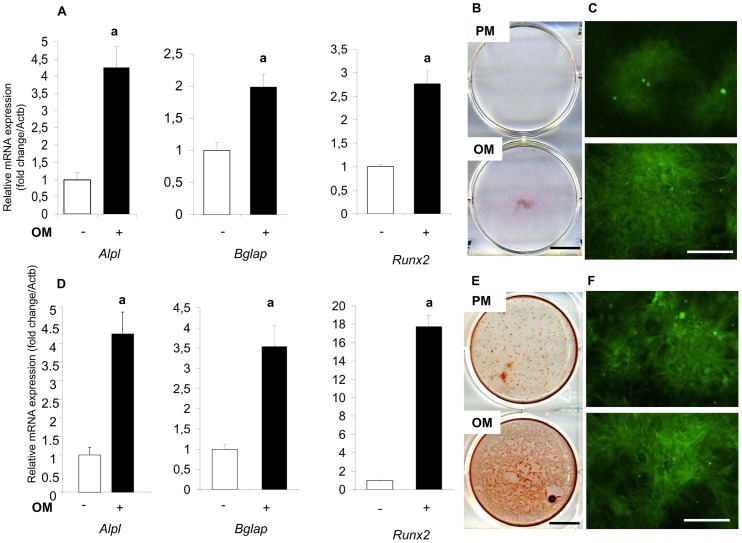
Osteogenic differentiation of GFP-expressing rat bone marrow-derived MSC at 14 and 28 days. Cells were grown for 14 (panels A, B and C) or 28 days (panels D, E and F) in osteogenic medium (OM) or proliferative medium (PM). A and D) Expression of the osteogenic marker genes *Alpl, Bglap*, and *Runx2* was investigated by real-time PCR. Results are expressed as relative expression level compared with the control condition in the absence of OM. a: p<0.05. B and E) Calcium deposition was investigated by Alizarin Red staining as described in Materials and Methods. Bar: 1000 µm. C and F) Fluorescence microscopy assessment of GFP-expressing cells was investigated in OM or PM. Bar: 500 µm.

A key characteristic of MSC is their ability, with adequate stimuli, to differentiate into multiple lineages, such as osteogenic and adipogenic cell lines. For osteogenesis, MSC were grown in a monolayer for a 14 and 28-days period in OM as described in Materials and Methods. Expression levels of osteogenic markers were investigated by real-time PCR at Days 14 and 28. At day 14, the relative expression levels of osteogenic genes (*Alpl, Bglap*, and *Runx2*) for cells grown in OM showed a respective 4, 2, and 2.5 fold increase compared to the non-OM control (Fig. 2A). At day 28, the relative expression levels of osteogenic genes (*Alpl, Bglap*, and *Runx2*) for cells grown in OM showed a respective 4, 3.5, and 18 fold increase compared to the non-OM control (Fig. 2D). Osteogenic differentiation was also evaluated by calcified matrix deposition through Alizarin Red staining. Alizarin Red-positive staining was detected as early as Day 14 and increased until Day 28 when OM was present (Fig. 2B and E). Interestingly, MSC grown in proliferative medium also showed some positive staining at Day 28, but less than the committed cells. GFP expression was preserved throughout the various passages and during cell differentiation (Fig. 2C and F).

**Figure 3:**
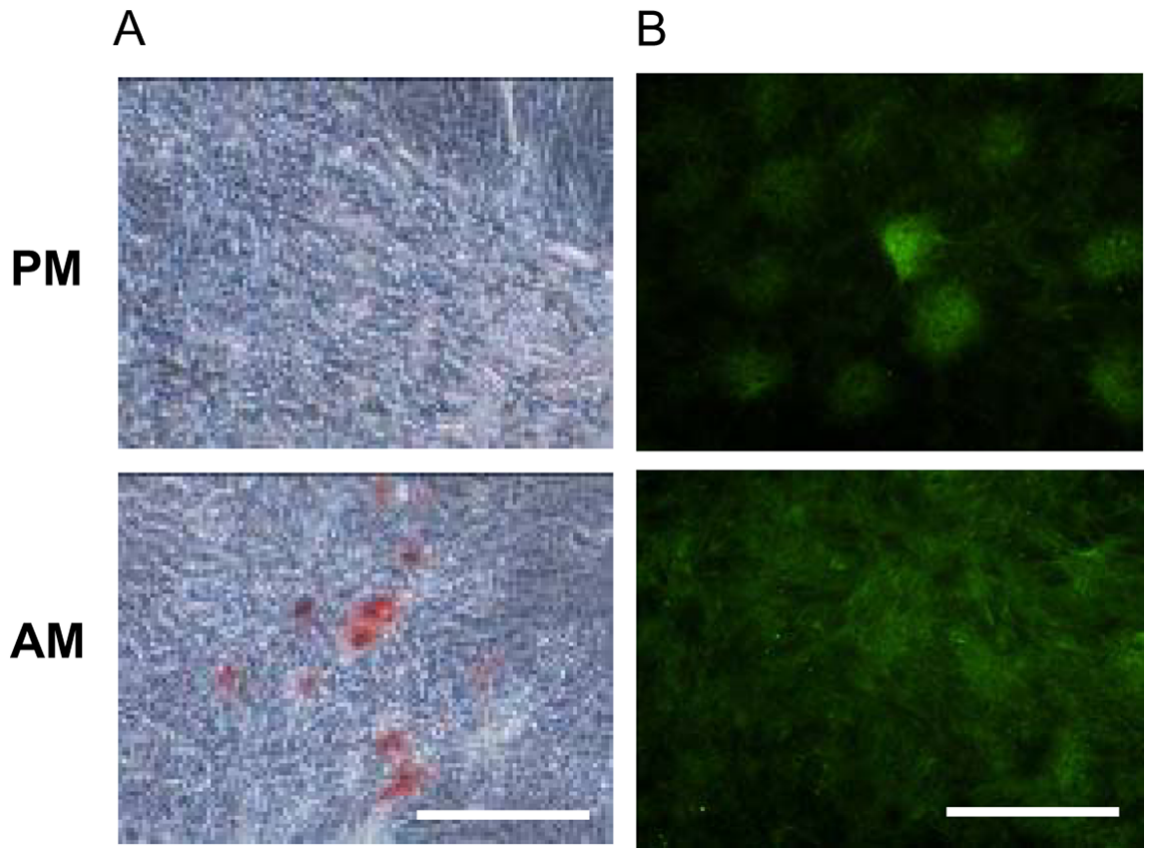
Adipogenic differentiation of GFP-expressing rat bone marrow MSC. Cells were grown in a monolayer for a 14-day period in adipogenic medium (AM) or proliferative medium (PM). A) Adipogenic differentiation was evaluated based on the production of neutral lipid containing vacuoles demonstrated by Oil red O staining. Bar: 500 µm. B) Fluorescence microscopy assessment of GFP-expressing cells was investigated in AM or PM. Bar: 500 µm.

For adipogenesis, MSC were grown in a monolayer for a 14-day period in the presence of adipogenic medium as described in Materials and Methods. The expression of the adipogenic markers *Pparγ* and *Lep* was investigated by real-time PCR. Whereas transcripts coding for *Pparg* and *Lep* were detected in adipogenic medium, their expression level remained undetectable in proliferative medium (data not shown). Adipogenic differentiation was also assessed using Oil red O staining through the appearance of neutral lipid containing vacuoles. Oil red O-positive staining was detected at Day 14 for cells grown in the presence of adipogenic medium compared with cells grown in proliferative medium (Fig. 3A). GFP expression was once again preserved throughout the different passages and during cell differentiation (Fig. 3B).

### Cell attachment, viability and osteogenic differentiation onto the CaP biomaterials for MSC Cult OM and PM conditions

**Figure 4:**
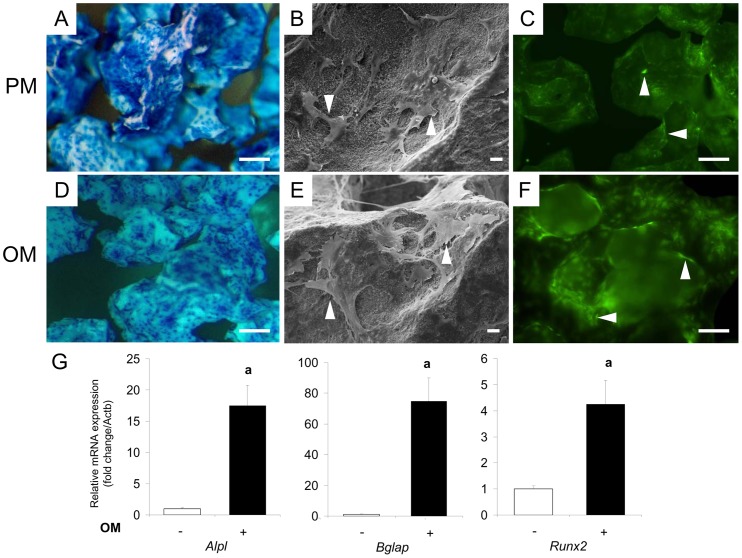
Adhesion and osteogenic differentiation of GFP-expressing transgenic rat bone marrow-derived MSC on BCP granules. At Day 14 in proliferative (PM; A, B and C) and osteogenic (OM; D, E and F) medium. A and D) Cell adhesion was investigated with methylene blue staining. Cells appeared in deep blue. Bar: 100 µm. B and E) Cell morphology was assessed on the surface of biomaterial by SEM after fixation and dehydration (arrow head). Bar: 20 µm. C and F) Microscopic observation of fluorescent GFP-expressing cells on BCP granules (arrow head). Bar: 50 µm. G) Expression of the osteogenic marker genes *Alpl, Bglap*, and *Runx2* was investigated by real-time PCR at the time of implantation (Day 14). Results are expressed as relative expression level compared with the control condition in the absence of OM. a: p<0.05.

To assess MSC attachment potential before implantation, methylene blue staining and SEM were performed (Fig. 4). We observed that cells grown in osteogenic or proliferative medium adhered to the surface of BCP particles in similar proportions. Cells were spread over the entire surface of material, but were observed preferentially in the concave areas of the BCP (Fig. 4A and D). When analyzing the BCP surface via SEM, we observed that cells exhibited mainly a branched or spindle morphology (Fig. 4B and E). In addition, the viability of attached GFP-expressing cells was verified using fluorescence microscopy for both the osteogenic and proliferative conditions (Fig. 4C and F). These results also indicated that cells grown in the different conditions expressed GFP before implantation. For osteogenesis, MSC were grown at the surface of BCP particles for a 14 days period in OM as described in Materials and Methods. Expression levels of osteogenic markers were investigated by real-time PCR at Day 14. At Day 14, the relative expression levels of osteogenic genes (*Alpl, Bglap*, and *Runx2*) for cells grown in OM showed a respective 17, 70, and 4 fold increase compared to the non-OM control (Fig. 4G).

### Histomorphometric and SEM assessment

**Figure 5:**
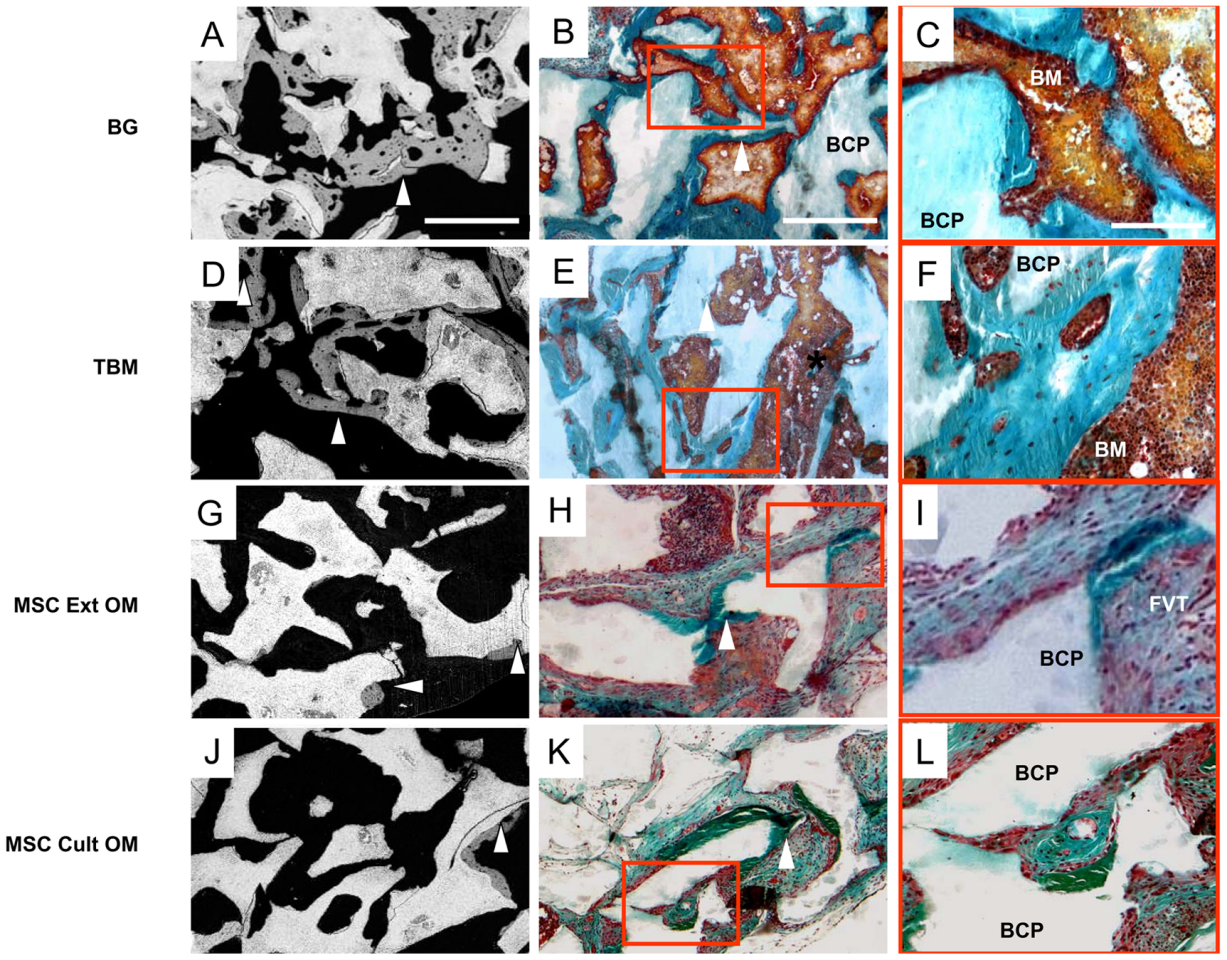
Ectopic bone formation at 8 weeks. Newly formed bone was assessed by SEM in samples embedded in GMMA (A, D, G and J). BCP granules appeared in white, non-calcified tissues in black, and bone in grey (arrow head). Bar: 500 µm. Histological study of newly formed bone and tissues surrounding the material after Goldner trichrome staining as described in Materials and Methods (B, E, H and K). Bone appeared in green (arrow head) BCP: biphasic calcium phosphate. Bar; 500 µm. Magnification of region of interest (red rectangle) showing the surrounding tissues (C,F, I and L). Bone marrow niche were observed only in BG and TBM conditions. In MSC Ext OM and MSC cult OM conditions, fibrovascular tissue was only observed between the BCP granules and bone. BM: bone marrow, FVT: fibrovascular tissue. Bar: 150 µm.

The MSC-, BG-, and TBM-loaded ceramics were implanted subcutaneously in nude mice, and revealed inconsistent osteogenic results after eight weeks. Histological observations were consistent with the SEM analysis, and new bone formation was only identified in four groups (BG, TBM, MSC Ext OM, and MSC Cult OM) (Fig. 5 A, D, G and J). In particular, abundant bone formation was seen in BG and TBM implants (Fig. 5 A and D), with woven bone over the ceramic surfaces. In the vicinity of granules, the bone connected individual particles with trabecula (Fig. 5 B and E). Moreover, the bone-lined pores were also filled with accompanying blood vessels. A rich medullar environment, containing hematopoietic progenitors and adipose tissue, was observed between the BCP granules and the bone (Fig. 5 C and F). In contrast, the MSC Ext OM and MSC Cult OM implants showed the least amount of bone formation, which was detected only within the peripheral pores of the implant (Fig. 5 H and K). Mostly loose connective tissue and vascular elements were present in the porous structure of these implants (Fig. 5 I and L). The other implants (granules alone, cells alone and MSC grown in proliferative medium loaded-BCP) failed to form bone. Almost all pores were filled with fibrovascular connective tissue. Conditions in which no bone was detected are not shown.

### Assessment of new bone formation

**Figure 6:**
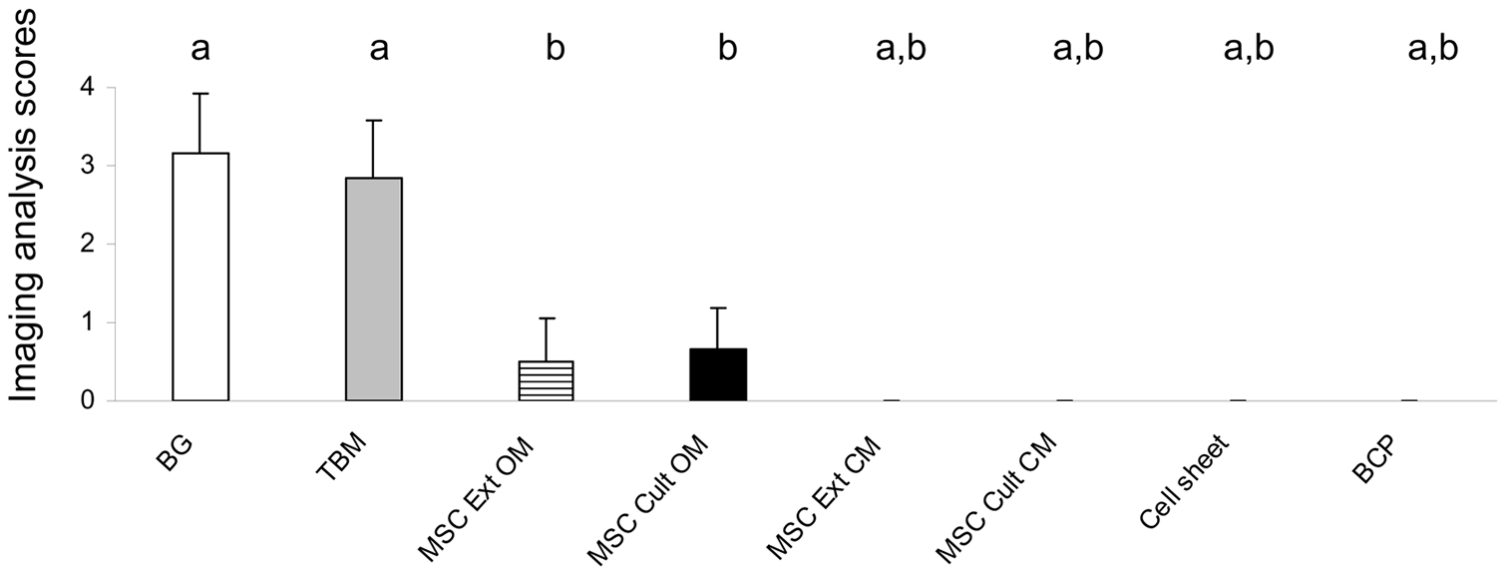
Ectopic bone formation scored at 8 weeks. Data are represented as mean ± S.D. a: *p*<0.05 as compared to MSC Ext OM. b: *p*<0.05 as compared to TBM. BG: bone graft, TBM: Total bone marrow, MSC Ext OM/MSC Ext PM: Mesenchymal stem cells grown in osteogenic medium/proliferative medium then extemporaneously loaded onto BCP granules. MSC Cult OM/MSC Cult PM: MSC loaded onto BCP granules then grown in osteogenic/proliferative medium. BCP: biphasic calcium phosphate alone.

Figure 6 summarizes the results of bone formation analysis using histological score. Bone formation was observed in only four groups (BG, TBM, MSC Ext OM, and MSC Cult OM). Implants with BG or extemporaneously blended TBM with BCP exhibited the highest amount of bone formation. However, implants that required- two or three-dimensional cell culture presented with limited osteogenesis.

### Tracking of GFP-labeled cells

**Figure 7:**
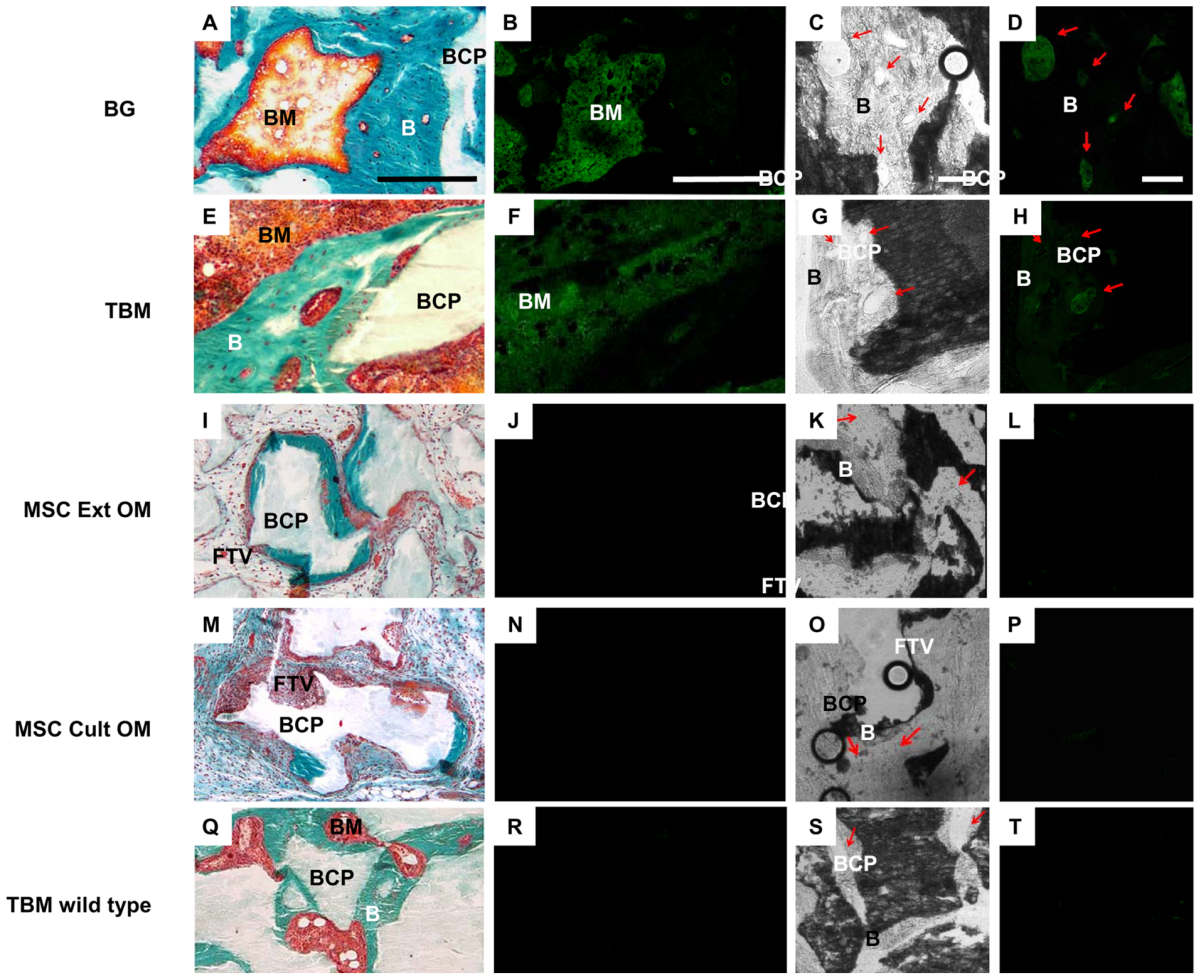
“*In vivo*” tracking of donor cells. Goldner trichrome staining (A, E, I, M and Q). B: bone, BCP: biphasic calcium phosphate, BM: bone marrow, FVT: fibrovascular tissue. Bar: 250 µm. Green fluorescence of GFP retrieved in subcutaneous implants (B, F, J, N and R). Nude mice implanted with non-GFP BM were used as negative controls (TBM wild type). Bar: 250 µm. Transmitted light showing vessels in connective tissues surrounding the BCP granules or in newly formed bone (red arrow) (C, G, K, O and S) Bar: 100 µm. Fluorescent light showing vessels only in TBM and BG groups (red arrow) (D, H, L, P and T) Bar: 100 µm.

To track GFP-expressing cells within the bone forming implants after eight weeks, two contiguous sections were used for each implant. The first was stained with Goldner trichrome to localize the support material, the newly formed bone, the vessels, and the marrow environment (first column of Fig. 7 A, E, I, M and Q). The second section (without any staining) was observed first under transmitted light (not shown) to detect the same elements previously observed with Goldner staining, then under a 535 nm fluorescent light source (second column of Fig. 7 B, F, J, N and R). Next, the same section (without staining) was observed with transmitted light at a higher magnification to detect vessels in newly formed bone or in the peripheral tissue (third column of Fig. 7 C, G, K, O and S). This higher magnification was also used for fluorescence imaging (fourth column of Fig. 7 D, H, L, P and T). Nude mice implanted with non-GFP wildtype BM were used as negative controls (Fig. 7 Q–T). Under fluorescent light, only the groups containing GFP-expressing BG (Fig. 7 B and D), or TBM (Fig. 7 F and H) were positive. Notably, the GFP signal was mainly observed in the marrow environment. A weak signal was also observed within the vessels present in the newly formed bone (Fig. 7 D and H). No signal was detected in osteocytes or osteoblasts. The groups containing GFP-expressing MSC (MSC Ext OM and MSC Cult OM) exhibited a weak signal, mainly in the loose fibrovascular tissue and vessels surrounding the particles. No signal was detected in the vessels or osteoblasts present in the newly formed bone (Fig. 7 J, L, N and P). As expected, non-GFP wild type bone marrow implants did not exhibit any GFP signal in the rich marrow environment, nor in the vessels of the newly formed bone (Fig. 7 R and T).

## Discussion

For bone repair, the grafting of fresh whole BM, with or without CaP biomaterials, is simple, safe, inexpensive, and well accepted. In fact, it has been proven to be effective in a wide range of applications including orthopedic surgery for posterolateral spinal fusion [41], [42] or tibial fracture healing [43], [44]. However, despite studies demonstrating the effectiveness of this technique for bone repair, it is not yet considered a gold standard. Indeed, the results obtained with BM grafting suffer from a lack of reproducibility, which is likely related to the poor number of MSC in BM aspirations [45]. It has been previously observed that human BM aspiration contains approximately 1 stem cell per 1.10^5^ nucleated cells in adults [46]. Although the optimal number of MSC required for efficient CaP biomaterial-associated bone formation has not been established and could range between 3.5.10^3^ to up to 7.5.10^4^ cells per mg of material [25], [29], [47]–[50], it is generally believed that increasing the number of grafted MSC will result in better bone formation [33]. This is likely related to the crucial role of MSC in osteoinduction [51].

In this context, it was recently suggested that increasing the number of grafted cells by tissue engineering strategies, which involve *in vitro* culture of MSC on the surface of CaP biomaterials, could make the production of engineered bone constructs possible. Such *in vitro* engineered hybrid constructs have been used in a large number of preclinical animal studies and have provided proof of efficiency [23], [50]. This strategy has also been tested in humans for bone regeneration [36], [37], [52], [53], but unfortunately failed to demonstrate a relevant efficiency when compared to BG. Also, it raises strong concerns regarding the regulatory framework governing the use of cell-based therapies and the complexity/cost of cell therapy.

Learning more about the efficacy of the various bone formation methods is necessary for addressing any uncertainties surrounding their clinical use. Thus, we were interested here in determining in an “all-in-one” study the most relevant bone engineering strategy. For this, we compared the effectiveness of eight bone regeneration procedures head-to-head in an ectopic model of osteoinduction. We selected methods that had previously demonstrated *in vivo* osteogenic properties in animal models or humans (i.e., CaP-assisted BG, extemporaneous association of fresh TBM with CaP granules, and several MSC-based bone tissue engineering strategies).

CaP-assisted BG, or bone expansion, was used as a positive control in our studies since it has been shown to promote the formation of well-organized bone in humans [35], [54], [55]. As a negative control, CaP biomaterials were grafted alone, as they are known to have little to no osteoinductive potential in subcutis ectopic sites [33], [56], [57]. Also, we examined a condition using cells alone, which consisted of high density committed MSC (known as cell sheets). The *in vivo* osteogenic potential of sheets was previously described [39]. Unprocessed fresh BM was also tested in combination with CaP following a procedure previously described in animal and human studies [14], [29]. Finally, several combinations of grown MSC and CaP were evaluated to test parameters influencing MSC-mediated bone formation *in vivo*. For this, we focused on (i) cell commitment towards the osteogenic lineage; (ii) preculture of cells in contact with CaP; and (iii) density of implanted cells [36]–[38].

The need for cells to be committed towards osteogenic differentiation before implantation is still a large matter of debate. Although in preclinical animal experiments it is unclear whether an osteogenic commitment of MSC is a prerequisite for *in vivo* bone formation [49], [57]–[59], most clinical studies in humans have been performed with precommitted osteogenic cells [37], [52], [53], [60].

To further determine whether osteogenic commitment was required for bone formation, we compared the ability of MSC precommitted or not by an OM [30] to form bone *in vivo*. In our experimental conditions, bone formation was only found when MSC were precommitted into the osteogenic lineage, as evidenced by our *in vitro* data. These results are consistent with those of previous studies [37], [52], [53], [60], and confirm that committed osteogenic MSC are able to promote bone formation.

Among the numerous bone engineering strategies used for human clinical studies, the three-dimensional culture of cells onto the surface of CaP biomaterials before implantation is probably the most widely used [37], [53], [60]. This culture system allows MSC, committed or not, to adhere to the CaP scaffold and produce early extracellular matrix [47]. The resulting hybrid bone construct is easy to manipulate and demonstrates osteogenic potential in human applications [37], [53]. Nonetheless, this complex co-culture process is time consuming, requires many controls, and exhibits safety and cost concerns for human application.

To simplify the procedure, cells may also be expanded in two-dimensional conditions without any CaP scaffold. After proliferation and possibly osteogenic differentiation, cells can then be combined with the scaffold and subsequently implanted. Indeed, this technique has demonstrated efficacy not only in immunocompromised mice and large animals [33], [49], [58], [61], but also in humans [21], [36], particularly when MSC were osteogenically differentiated. Consistent with the literature data [62], [63], our study revealed that only differentiated MSC, regardless of culture dimensionality, promoted new bone formation at eight weeks. However, the amount of bone formed with MSC was always lower as compared to the group with TBM.

Interestingly, few studies have compared the ability of CaP materials associated with MSC or unprocessed BM to form bone *in vivo*. In large and small animal models of healthy bone critical-sized defects, MSC promoted more bone formation than BM grafting [18], [49]. On the contrary, BM grafting was found to be the most efficient strategy to repair critical-sized defects in hypotrophic irradiated bone [29]. These studies seem to be contradictory. However, these data led us to hypothesize that whereas MSC are able to induce bone formation in healthy orthotopic sites [18], [49], they might lose osteogenic capabilities in hypotrophic or unfavorable environments (e.g., low nutrients or low oxygen tension) [29], [64].

In line with this hypothesis, converging data have recently reported that grafted MSC die early after *in vivo* implantation [25], [64]. In fact, it was convincingly demonstrated that grafted human MSC do not survive more than three weeks after subcutaneous implantation in nude mice [25]. In addition, Deschepper *et al*. recently suggested that death of subcutaneously grafted MSC on CaP biomaterials was not only due to hypoxia, but also glucose depletion [64]. Therefore, it seems reasonable to speculate that in our subcutaneous model of implantation, the amount of newly formed bone strongly depended on the survival rate of grafted cells. In order to address this issue, we performed a cell survival tracking experiment using GFP-labeled cells.

Our data revealed that after eight weeks of implantation in the TBM and BG groups, marrow and vessels found in the newly formed bone contained GFP-labeled cells. Even though it was not possible to clearly assign the GFP signal to osteoblasts and osteocytes within the *de novo* bone, these data suggested that grafted cells from TBM and BG groups contributed indirectly to the formation of these tissues. In contrast, when extemporaneous and grown GFP-labeled MSC were implanted, we failed to detect any GFP-labeled cells in the bone, adjacent vessels, or near BCP granules. While we cannot totally rule out the possibility that MSC might still be there but do not express GFP anymore at 8 weeks, it remains unlikely. Indeed, Remy *et al*., who first described the transgenic GFP-rat strain, previously implanted GFP-neural stem cells in striatum of rats. At 120 days, mature neurons expressed a GFP signal [28]. In our experimental conditions, one can therefore assume that most of the grafted MSC died early after implantation as previously speculated [25], [64]. Under these conditions, the residual low-level of bone formation could be a result of remaining MSC releasing some osteotrophic growth factors, which had initially influenced the osteogenic potential of host cells. In addition, MSC were probably not cultured in optimal conditions before implantation and thereby could not support a large bone formation. However, to carry out our comparative study, we had to make a choice between many available bone reparation strategies. Our choice was firstly based on the efficacy of such strategies demonstrating bone formation in animal or human; and secondly on their clinical feasibility with respect to the European regulatory frame. We thus emphasized on cell-based strategies with gradually increasing complexity to provide some realistic data to the clinicians and patients. Though we could have used bone stimulatory factors during the *in vitro* stage, like cytokines, transgenic approach, or bioreactors, but these strategies are currently not transposable in clinic in most of the EU countries and thereby will not be available for clinicians in a near future.

Taken together, these findings suggest that the newly formed bone observed in our study appeared to be a chimera derived both from the grafted and host cells. Similar observations have been previously reported in a model of osteoinduction by Goshima *et al*. using BM cell labeling [65]. Indeed, following subcutis implantation of BM blended CaP biomaterials, they observed that ectopic bone-resident osteoblasts and osteocytes were derived from the grafted cells during the early phase (21 days) and from the host cells in a secondary phase (by 56 to 84 days), with the donor derived bone being gradually replaced by bone derived from the host [66].

## Conclusions

The results of this study indicated that more significant bone formation could be obtained with TBM than with several BTE strategies. In addition, they revealed a trophic property of implanted BM, which displayed a capacity to survive and develop eight weeks after implantation. Owing to its simplicity and safety, this method could be performed by surgeons during a single procedure and without additional cost. The variability of BM aspiration quality from donor to donor represents the main limitation of both the TBM grafting and MSC culture [45], but contrarily to the TBM grafting that remains a basic procedure, MSC culture protocols could probably be largely improved in a near future. Nevertheless, in light of increasing regulatory constraints for clinical trials [67] and persistent scientific concerns regarding stem cells in bone regenerative medicine, BM grafting could represent a clinically relevant alternative to BG and BTE. Since ectopic mouse implantation represents a preliminary model in testing clinically relevant bone tissue engineering strategies, further studies are now necessary to assess this method in a bone defect model.
